# Delayed treatment of α5 GABAA receptor inverse agonist improves functional recovery by enhancing neurogenesis after cerebral ischemia-reperfusion injury in rat MCAO model

**DOI:** 10.1038/s41598-019-38750-0

**Published:** 2019-02-19

**Authors:** Wei-Ming He, Li Ying-Fu, He Wang, Yu-Ping Peng

**Affiliations:** 1 Neurosurgery, Nanfang Hospital, Southern Medical University, Guangzhou, 510515 P. R. China; 2 Neurosurgery, Affiliated Hospital of Inner Mongolia University for the Nationalities, Tongliao City, Inner Mongolia 028000 P. R. China; 3 Neurosurgery, First Hospital, Jia Mu Si University, Jiamusi, 154007 P. R. China

## Abstract

Development of effective therapeutics and treatment strategy to promote recovery after cerebral ischemia-reperfusion injury necessitates further understandings of the complex pathophysiology of ischemic stroke. Given that α5-GABA_A_R inhibition has been shown to be involved in functional recovery after stroke, the present study was designed to evaluate the effects of treatment timing of α5 GABA_A_R inhibition on post-middle cerebral artery occlusion (MCAO) functional recovery. To this end, we examined the effects of L655,708 (α5 GABA_A_R inverse agonist) treatment at 3 or 7 days post-ischemia on apoptosis and neurogenesis in the peri-infarct region, brain infarction size, as well as modified neurological severity score (mNSS) and rotarod test time in rats. Consistent with previous reports, we found that when the treatment of L655,708 was initiated at post-MCAO day 3, it did not alter the functional recovery in rats. However, when the treatment of L655,708 was initiated at post-MCAO day 7, it demonstrated beneficial effects on functional recovery in rats. Interestingly, this phenomenon was not associated with altered brain infarction size nor with changes in brain cell apoptosis. However, we found that delayed treatment of L655,708 at post-MCAO day 7 significantly increased neurogenesis in peri-infarct zone in rats. These results suggested that removing α5 GABA_A_R-mediated tonic inhibition after cerebral ischemia-reperfusion injury may be an effective therapeutic strategy for promoting functional recovery from stroke.

## Introduction

Ischemic stroke is a common type of stroke and is a major cause of neurological disability worldwide^1^. Restoration of blood flow following ischemic stroke can be achieved by means of thrombolysis or mechanical recanalization. However, reperfusion may exacerbate the injury initially caused by ischemia. Thus, it will bring significant societal and health impacts by preventing cerebral ischemia-reperfusion injury and/or promoting function recovery to reduce dependence and improve the quality of life of stroke survivors. However, development of effective therapeutics and treatment strategy necessitates further understandings of the complex pathophysiology of ischemic stroke.

Previous studies suggested that recovery of function following acute brain injury to the cortical regions can be promoted by the reduction of brain GABA availability^2^. These findings suggest targeting of GABA receptors that mediate tonic inhibition could be a novel strategy to promote post-stroke functional recovery. However, recent studies indicated that GABA receptor, specifically α5-GABA_A_R, may play distinct roles in a time-dependent manner after stroke during the recovery. Specifically, in a cortical stroke model, treatment with α5-GABA_A_R inverse agonist immediately after stroke increased size of the cortical lesion^3^. However, the same study also demonstrated that treatment with α5-GABA_A_R inverse agonist at 3 days after stroke significantly promoted functional recovery in mice^3^. However, the neurobiological mechanisms underlying this phenomenon are still not clear. In fact, neuronal plasticity can occur after stroke, particularly in the peri-infarct zone that is adjacent to the region damaged by the stroke^4^. In addition to irreversible neuronal damage, brain ischemia can trigger apoptosis^5^ and induce neurogenesis^6,7^, which can contribute to functional recovery after stroke^5,6^. Hence, α5-GABA_A_R inverse agonist induced functional recovery may be resulted from a complex interplay among apoptosis and neurogenesis following brain ischemia.

Therefore, in the present study, we tested the hypothesis that delayed treatment with α5-GABA_A_R inverse agonists can enhance functional recovery after MCAO-induced brain ischemia-reperfusion injury in rodents. To this end, we examined the effects of L655,708 treatment at 3 or 7 days post-ischemia on apoptosis and neurogenesis in the peri-infarct region, brain infarction size, as well as modified neurological severity score (mNSS) and rotarod test time in rats.

## Materials and Methods

### Animals

Adult male Sprague–Dawley (SD) rats weighing between 250 and 300 g were ordered from Shanghai Experimental Animal Center. Upon arrival, animals were housed in a 12–12-hour light-dark cycle environment. The room temperature was controlled at 25 °C, and animals were allowed to access food and water *ad libitum* throughout the study. The use of animal was approved by the Institutional Animal Care and Use Committee of National Institute for Viral Disease Control and Prevention, China Center for Disease Control and Prevention, Beijing, and all animal experiments were conducted in accordance with the Guide for the Use of Laboratory Animals.

### Transient middle cerebral artery occlusion (MCAO) model and animal treatment

Rats were under isoflurane anesthesia. We exposed the right middle cerebral artery (MCA) through a cranial burr hole, which was located approximately 2.5 mm lateral and 2.0 mm posterior to the bregma. The blood flow of MCA was monitored using a Laser Doppler flowmetry (Moor Instruments Inc., Wilmington, USA). The blood flow of MCA was above 500 min/div before MCAO procedure. We then exposed both the right common carotid artery (CCA) and internal carotid artery (ICA) via a neck midline incision, followed by the ligation of the pterygopalatine artery proximal to its branch. We then inserted a 3–0 nylon filament suture coated with poly-L-lysine (Sigma-Aldrich, Shanghai, China) into the right external carotid artery through the CCA and moved it up to the ICA at a distance of 20–25 mm to block the right MCA. As a result, the blood flow of MCA was decreased to less than 100 min/div (>80%). Blood flow from the right MCA was blocked for 15 minutes, followed by removing the suture for reperfusion (a return to >7% of the baseline within 10 min of suture withdrawal). Sham-operated rats received the same surgery process without the MCAO. The treatment with L655,708 (α5-GABA_A_R inverse agonist, 0, 1, or 5 mg/kg) was initiated at post-MCAO days 3 and 7. Rats received intraperitoneal injections of vehicle (i.e., saline) or L655,708 once daily for 4 consecutive days. Sham-operated rats received no treatment, and a separate group of MCAO rats received vehicle injections once daily for 4 consecutive days starting at post-MCAO day 1.

### Modified neurological severity score (mNSS) assessment and rotarod test (RRT)

Modified neurological severity score (mNSS) assessment was performed at post-MCAO day 14 by a well-trained research associated who was blinded to the experimental conditions. The mNSS scale provides a behavior deficit score, reflecting motor, sensory, balance, and reflex functions as described previously^8,9^. A normal score was represented by 0 and the maximal deficit score was 18. The rotarod test was conducted in an apparatus (Harvard Apparatus, Holliston, MA, USA) consisted of a striated rod. In the present study, each rat was first trained for 3 days on a rotarod cylinder before testing, as described previously^10^. At post-MCAO day 14, rats were tested for the time performed during the rotarod test.

### Triphenyltetrazolium chloride (TTC) staining

We sectioned the rat brain from the frontal pole into six slices with 2 mm thickness. The brain slices were stained with 2% 2,3,5-triphenyl tetrazolium chloride (Sigma-Aldrich, Shanghai, China) for 15 minutes. The ischemic infarction area was stained white, and the non-ischemic infarction area was stained red. We took the images of each brain slice (n = 8), and the ratio of the ischemic area to the ipsilateral hemisphere area (I/H ratio) was calculated using total ischemic area divided by total ipsilateral hemisphere area with NIH Image J.

### Tissue preparation for histochemistry

All rats were sacrificed after the completion of behavioral function tests at day 14 after MCAO (n = 8 per group at each time point) with an overdose of 10% chloral hydrate. Rats were then perfused transcardially with 0.9% saline at 4 °C followed by 4% paraformaldehyde in phosphate buffer (0.1 mol/L, pH 7.4) based on the methods described previously^11^. Rat brains were quickly removed, fixed in 4% paraformaldehyde for at least 8 hours at 4 °C, and then restored in 20% and 30% sucrose. 10-μm thick coronal sections were prepared using a cryostat (CM1900; Leica, Shanghai, China) from bregma + 4.0 to −6.0 mm. Tissues were then used for subsequent immunoflourescence staining or TUNEL staining.

### Immunofluorescence staining

Brain sections were first pretreated with citrate buffer (0.01 mol/L, pH 6.0) for 5 minutes at 85 °C, followed by incubation with 5% normal goat serum for 1 hour at room temperature. Sections were then incubated with anti-Ki67 (rabbit monoclonal antibody to Ki67, 1:500; Cat# ab16667 RRID: AB_302459; Abcam, Shanghai, China) and anti-NeuN (rabbit monoclonal antibody to NeuN, 1:500; Cat# ab190565, RRID:AB_2732785; Abcam, Shanghai, China), overnight at 4 °C. Sections were then rinsed in phosphate-buffered saline 3 times, followed by incubation with rabbit secondary antibody (anti-rabbit IgG, 1:1000, Cell Signaling, Shanghai, China) for 1 hour at room temperature. Fluorescence signals were then detected under a microscope (BX51; Olympus, Japan). Negative control was conducted by incubating sections with PBS instead of primary antibodies. No positive signals were shown in negative control.

### Terminal deoxynucleotidyl transferase dUTP nick end labeling (TUNEL)

TUNEL staining was performed as previously described^12^ using recombinant terminal deoxynucleotidyl transferase (TdT), following the manufacturer’s instructions (Roche, Shanghai, China). TUNEL signal was detected with Alexa 488-labelled secondary antibody for streptavidin. Cell nuclei were stained with DAPI. Negative controls of TUNEL staining were performed without TdT.

### Image analysis and quantification

Image-Pro Plus image analysis software (Media Cybernetics, Silver Spring, MD, USA) was used for images data analysis. Data analysis was conducted by a researcher who was blinded to the experimental conditions. We focused on the region in the peri-infarct zone, which was immediately outside the infarct zone. To count TUNEL/Ki67-immunopositive cells, 8 consecutive sections from bregma 0.20 to −2.20 mm with 240-μm interval were included for analysis. The number of TUNEL/Ki67-immunopositive cells in the peri-infarct region was counted in 3 separate, non-overlapping fields (425 μm × 320 μm) under ×400 magnification. The average cell number per field was used for each section, the average cell number of all the sections was used as the final cell number per rat^13^.

### Statistical analysis

All data were presented as mean ± SEM. Statistical analysis was performed using SPSS 18.0 for Windows (SPSS Inc, Chicago, IL, USA). One-way ANOVA or t-test were used to evaluate mNSS, rotarod time, Ki67-positive and TUNEL-positive cell numbers for each treatment starting time groups. *p < 0.05 is considered to be significant when comparison was made.

## Results

### Effects of delayed treatment of L655,708EA on MNSS in MCAO rats

**Figure 1:**
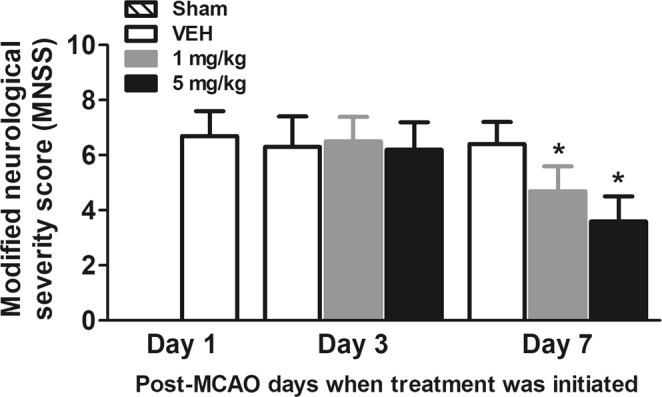
Effects of delayed treatment of L655,708EA on MNSS in MCAO rats. *Asterisks* represent the significant difference from vehicle (n = 8/group; p < 0.05).

We evaluated the effects of L655,708 treatment on neurological function using Modified Neurological Severity Score (MNSS) scale (Fig. 1). When the treatment was initiated at post-MCAO day 3 for 7 consecutive days, the MNSS of vehicle-treated rats was 6.3 ± 1.1. Similarly, the MNSS of 1 mg/kg or 5 mg/kg L655,708-treated rats were 6.5 ± 0.9 and 6.2 ± 1.0, respectively. In contrast, when the treatment was initiated at post-MCAO day 7 for 7 consecutive days, L655,708-treated rats exhibited significantly (p < 0.05) lower MNSS. Specifically, the MNSS of vehicle-treated rats was 6.4 ± 0.8. However, the MNSS of 1 mg/kg or 5 mg/kg L655,708-treated rats were 4.7 ± 0.9 and 3.6 ± 0.9, respectively.

### Effects of delayed treatment of L655,708EA on rotarod test time in MCAO rats

**Figure 2:**
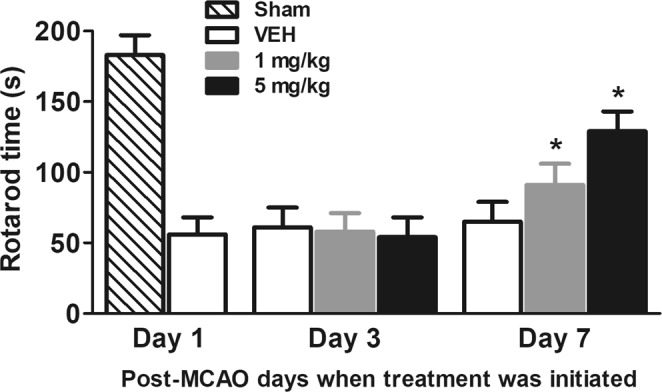
Effects of delayed treatment of L655,708EA on rotarod test time in MCAO rats. *Asterisks* represent the significant difference from vehicle (n = 8/group; p < 0.05).

The rotarod test times were higher in the sham group (183.2 ± 14.1 s) than in vehicle-treated MCAO control group (56.5 ± 12.4 s; Fig. 2). When the treatment was initiated at post-MCAO day 3 for 7 consecutive days, the rotarod test time of vehicle-treated rats was 61.3 ± 14.2. Similarly, the rotarod test time of 1 mg/kg or 5 mg/kg L655,708-treated rats were 58.5 ± 12.9 and 54.6 ± 13.7, respectively. In contrast, when the treatment was initiated at post-MCAO day 7 for 7 consecutive days, L655,708-treated rats exhibited significantly (p < 0.05) lower rotarod test time. Specifically, the rotarod test time of vehicle-treated rats was 65.2 ± 14.7. However, the rotarod test time of 1 mg/kg or 5 mg/kg L655,708-treated rats were 90.9 ± 15.2 and 128.8 ± 13.7, respectively.

### Effects of delayed treatment of L655,708 on the volume of the infarct area

**Figure 3:**
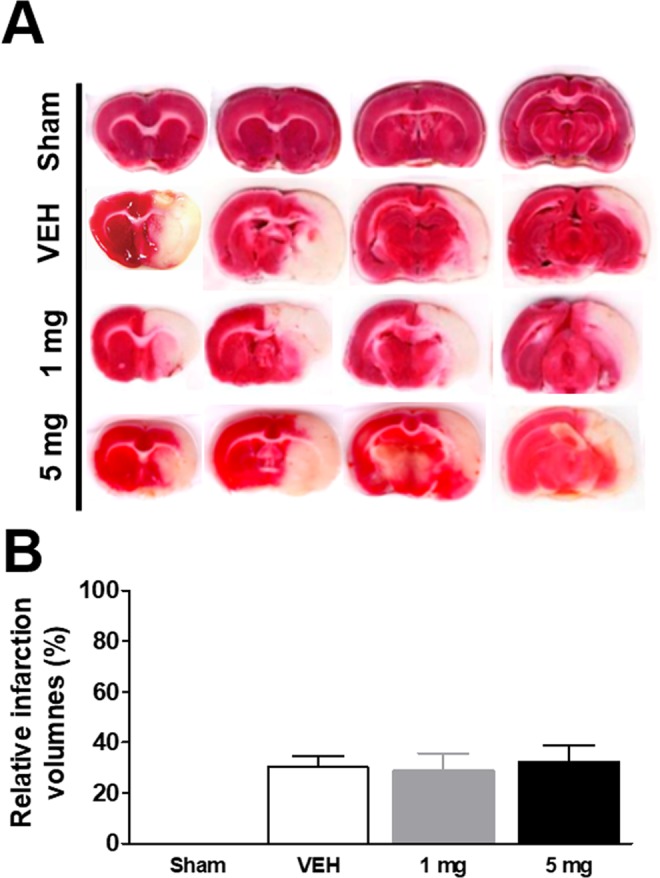
Effects of delayed treatment of L655,708 on the volume of the infarct area. The treatment was initiated at post-MCAO day 7, and we collected rat brains at post-MCAO day 14 (n = 8/treatment group).

To test the effects of treatment of L655,708 on the volume of the infarct area, when the treatment was initiated at post-MCAO day 7, we collected rat brains at post-MCAO day 14, and carried out TTC staining (Fig. 3A). We found that the relative infarct volume was 30.1 ± 4.6% in vehicle-treated group at post-MCAO day 14 (Fig. 3B). The relative infarct volumes of 1 mg/kg or 5 mg/kg L655,708-treated rats were 28.5 ± 7.1% and 32.1 ± 6.7% respectively, at post-MCAO day 14 (p = 0.31; Fig. 3B). These results indicated that delayed treatment of L655,708 after MCAO did not alter the volume of the infarct area.

### Effects of delayed treatment of L655,708 on apoptosis in the peri-infarct region

**Figure 4:**
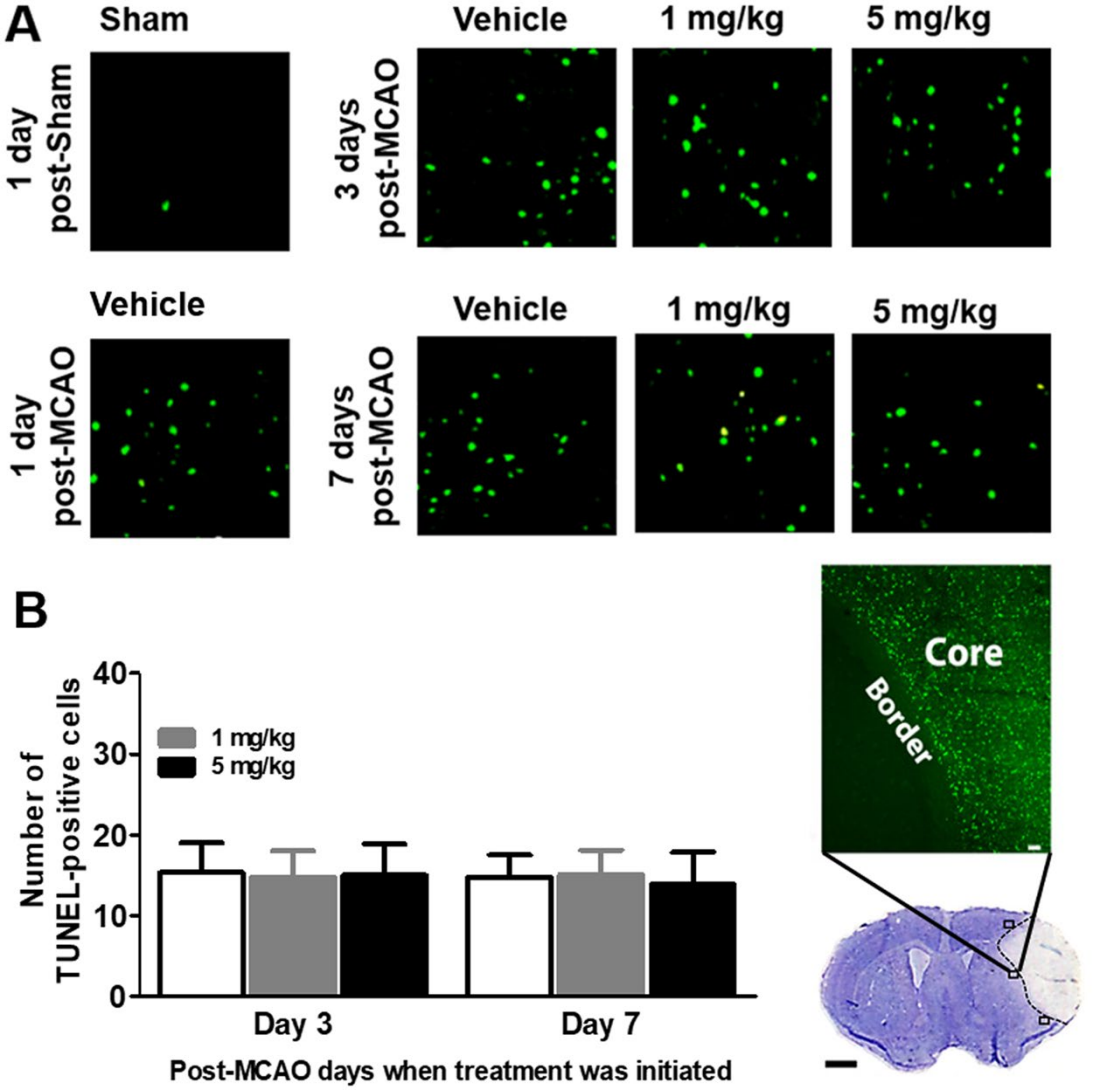
Effects of delayed treatment of L655,708 on apoptosis in the peri-infarct region. TUNEL assays were performed to examine the cell apoptosis in the peri-infarct region at post-MCAO day 14 (n = 8/group). Representative images of cresyl violet-stained brain sections (scale bar = 1 mm) and terminal deoxynucleotidyl transferase-mediated dUTPnick end-labeling (TUNEL)-stained section (scale bar = 50 µm). Squares in the cresyl violet-stained section are the three regions used to quantify TUNEL + cells. TUNEL-stained section shows the infarct border. Scale bar = 1 mm.

We conducted TUNEL assays to examine the cell apoptosis in the peri-infarct region at post-MCAO day 14. We first found that TUNEL-positive cells were limited in sham-operated group (Fig. 4A,B) and in the contra-lateral hemisphere (data not shown). In contrast, MCAO robustly increased the number of TUNEL-positive cells in peri-infarct region at post-MCAO day 14 (Fig. 4A,B). MCAO rats that received vehicle treatment initiated at post-MCAO day 1, 3, and 7 once daily for 7 consecutive days exhibited increased number of TUNEL-positive cells in the ipsilateral hemisphere (Fig. 4A,B). Furthermore, neither 1 mg/kg nor 5 mg/kg of L655,708 treatment initiated at post-MCAO day 3 and 7 once daily for 7 consecutive days altered the number of TUNEL-positive cells in the ipsilateral hemisphere compared with vehicle (Fig. 4A,B).

### Effects of delayed treatment of L655,708 on neurogenesis in the peri-infarct region

**Figure 5:**
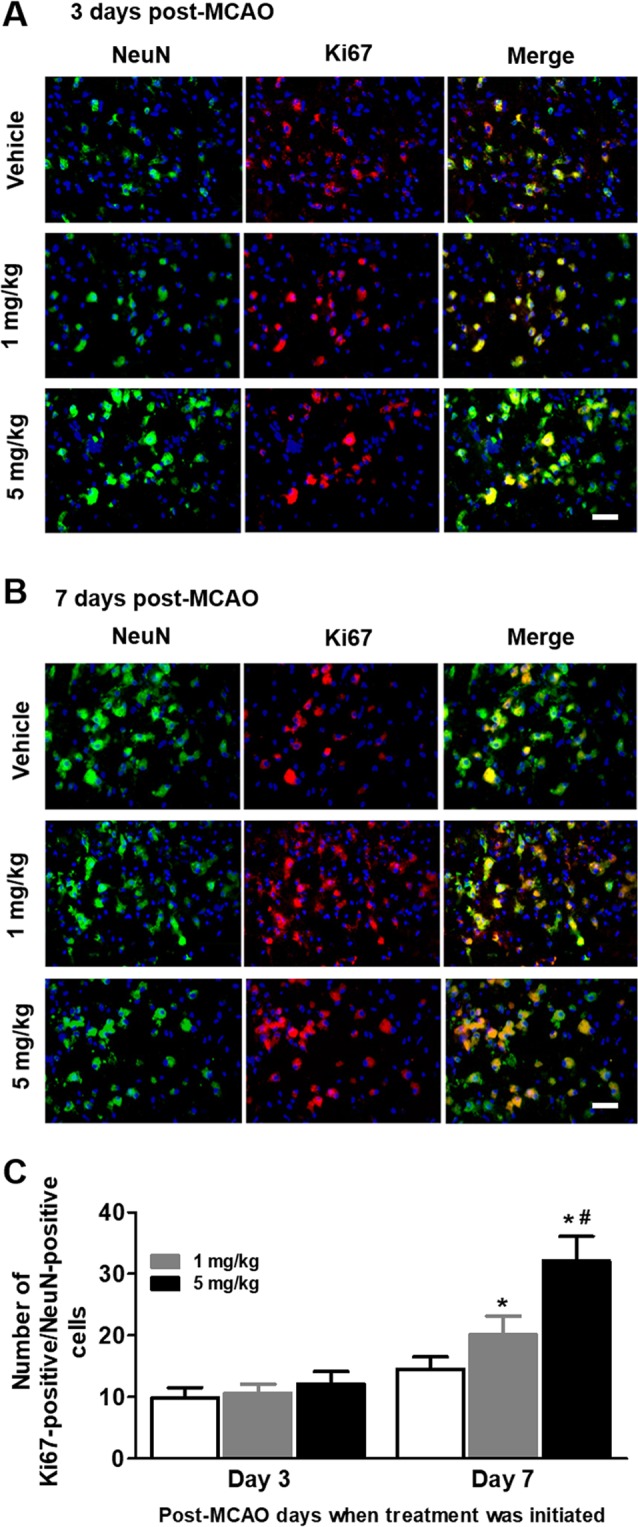
Effects of delayed treatment of L655,708 on neurogenesis in the peri-infarct region. Ki67, a proliferative marker, and NeuN, a neuronal marker, were used to evaluate neurogenesis in the peri-infarct region. The nuclei were counter-stained with DAPI. *Asterisk* represents the significant difference from vehicle (n = 8/group; p < 0.05). *Pond* represents the significant difference from 1 mg group (n = 8/group; p < 0.05). Scale bar = 25 µm.

We used Ki67, a proliferative marker, and NeuN, a neuronal marker, to evaluate neurogenesis in the peri-infarct region. We first found that Ki67-positive cells were limited in sham-operated group and in the contra-lateral hemisphere (data not shown). In contrast, MCAO robustly increased the number of Ki67-positive cells in peri-infarct region at post-MCAO day 14. MCAO rats that received vehicle treatment initiated at post-MCAO day 1, 3, and 7 once daily for 7 consecutive days exhibited increased number of Ki67-positive/NeuN-positive cells in the ipsilateral hemisphere (Fig. 5A,B). Furthermore, 1 mg/kg or 5 mg/kg of L655,708 treatment initiated at post-MCAO day 3 and 7 once daily for 7 consecutive days significantly (p < 0.05) increased the number of Ki67-positive cells in the ipsilateral hemisphere compared with vehicle (p < 0.05; Fig. 5C).

## Discussion

The present study was designed the evaluate the effects of treatment timing of α5 GABA_A_R inverse agonist on post-MCAO functional recovery. Consistent with previous reports, we found that when the treatment with L655,708, selective α5 GABA_A_R inverse agonist, was initiated at post-MCAO day 3, it did not alter the functional recovery in rats. However, when the treatment of L655,708 was initiated at post-MCAO day 7, it demonstrated beneficial effects on functional recovery in rats. Interestingly, this phenomenon was not associated with altered brain infarction size nor with changes in brain cell apoptosis. However, we found that delayed treatment of L655,708 at post-MCAO day 7 significantly increased neurogenesis in peri-infarct zone in rats. These results suggested that removing α5 GABA_A_R-mediated tonic inhibition after stroke may be an effective therapeutic strategy for promoting functional recovery from stroke.

GABA, as a primary inhibitory neurotransmitter in central nervous system, has received increasing attention in ischemic brain injury^14,15^. Recent reports have demonstrated that increasing GABAergic synaptic transmission might display neuroprotective effects against brain ischemia-reperfusion injury^16,17^. GABA can activate three types of specific receptors: GABA_A_, GABA_B_ and GABA_C_ receptors^18^. Among them, GABA_A_ receptors play an important role in regulating the dynamic balance between neuronal inhibition and excitation^19^. Importantly, a previous study has shown that in a cortical stroke model, treatment with α5-GABA_A_R inverse agonist immediately after ischemia-reperfusion injury increased size of the cortical lesion^3^, suggesting that activation of α5-GABA_A_R may be protective. Hence, future studies will test whether activation of α5-GABA_A_R prior the occurrence of stroke can reduce subsequent ischemia-reperfusion injury.

Currently, physical rehabilitation is the only available therapy for patient with ischemic stroke to promote functional recovery, and no pharmacological therapy has been approved by the FDA for this indication^20,21^. Despite this situation, studies have demonstrated that limited capacity for functional recovery after stroke exist in the brain. These post-stroke neural repair process involves recovery of physiological functions in tissue adjacent to or connected with the stroke^22,23^, including changes in neuronal excitability that alter the brain’s representation of motor and sensory functions. Studies have shown that enhancing neuronal excitability in peri-infarct zone can have beneficial outcome in recovery. Specifically, it has been demonstrated that stimulation of peri-infarct cortex alters sensorimotor maps and improves use of affected limbs^22–25^. One possible mechanism underlying this phenomenon might involve altered GABAergic transmission. This is because GABAergic transmission plays a critical role in the changes of sensory maps during cortical development^26^, a phenomenon resembling the changes of sensorimotor maps that occur after stroke^27,28^. Furthermore, numerous studies have demonstrated that GABAergic neurotransmission play a fundamental role in neuronal excitability^29^. GABA_A_R-mediated cortical GABAergic signaling consist of both synaptic and extrasynaptic components. Tonically active extrasynaptic GABA_A_Rs is involved in controlling the excitability threshold of neurons^30–32^. Among these extrasynaptic GABA_A_Rs, α5 or δ-subunit-containing receptors are the majority^30–32^. Pharmacological inhibition and genetic knockdown of α5 GABA_A_R can enhance LTP and improve performance on learning and memory tasks^33,34^. These studies suggested that α5-GABA_A_R is critical for cellular excitability and plasticity. Recent studies showed that enhancing levels of cell-intrinsic neuronal activity could increase survival of new neurons. Therefore, it is likely that removing α5 GABA_A_R-mediated tonic inhibition after stroke that promoted functional recovery from stroke may involve changes in neuronal excitability in the peri-infarct cortex after stroke leading to enhance survival of new neurons. Hence, future studies will be necessary to clarify the role of neuronal excitability in the peri-infarct cortex after stroke in neurogenesis.

One of the important factors that should be considered for pharmacotherapy development is the timing of pharmacological treatment. It has been shown that activation of GABA_A_Rs at the onset of stroke can reduce stroke size^35^. Therefore, it is likely that inhibition of GABA_A_R at earlier time after ischemic stroke may increase cell death and stroke size. In fact, previous studies using L655,708 have shown that stroke volume was significantly increased in animals treated with L655,708 at the onset of stroke, but when the same treatment was introduced to rats with a 3-day delay, stroke volumes were similar between mice treated with vehicle and L655,708. These results clearly indicate there is a critical timeframe to introduce the therapeutic to allow for inhibition of tonic GABA_A_R: inhibition of tonic GABA_A_R too early would promote stroke damage, while delaying inhibition of tonic GABA_A_R would promote functional recovery without altering stroke size. Adding to this literature, our study further demonstrated that a longer delay (i.e., 7 days after stroke) before the introduction of L655,708 can promote functional recovery.

In summary, our study confirmed that α5-GABA_A_R in the peri-infarct zone plays a critical role in neurogenesis and functional recovery for ischemic stroke. These results may provide an important pharmacological target that allows for promoting recovery after stroke.
